# (*E*)-1-(2,5-Dimethyl-3-thien­yl)-3-(2-hy­droxy­phen­yl)prop-2-en-1-one

**DOI:** 10.1107/S1600536810031284

**Published:** 2010-08-11

**Authors:** Abdullah M. Asiri, Salman A. Khan, M. Nawaz Tahir

**Affiliations:** a The Center of Excellence for Advanced Materials Research, King Abdul Aziz University, Jeddah 21589, PO Box 80203, Saudi Arabia; b Department of Chemistry, Faculty of Science, King Abdul Aziz University, Jeddah 21589, PO Box 80203, Saudi Arabia; c Department of Physics, University of Sargodha, Sargodha, Pakistan

## Abstract

In the title compound, C_15_H_14_O_2_S, the dihedral angle between the aromatic rings is 8.46 –(8)°. The central enone group is planar (r.m.s. deviation = 0.0267 Å) and is oriented at a dihedral angle of 1.20 (9)° with respect to the benzene ring and at 8.27 (9)° with respect to the thio­phene group. In the crystal, the mol­ecules are linked into polymeric chains extending along the *b* axis due to inter­molecular O—H⋯O hydrogen bonding. An *S*(6) ring motif is formed due to a short intra­molecular C—H⋯O contact. C—H⋯π inter­actions involving a methyl group of the 2,5-dimethyl­thienyl group and the benzene ring are present. π–π inter­actions between the centroids of the benzene and heterocyclic rings [3.7691 (9) Å] also occur.

## Related literature

For background to chalcones and their biological activity, see: Bandgar & Gawande (2010 ▶); Domínguez *et al.* (2001 ▶); Hans *et al.* (2010 ▶); Kayser & Kiderlen (2001 ▶); Mojzis *et al.* (2008 ▶); Vogel *et al.* (2010 ▶). For related structures, see: Asiri *et al.* (2010*a* ▶,*b* ▶); For graph-set notation, see: Bernstein *et al.* (1995 ▶).
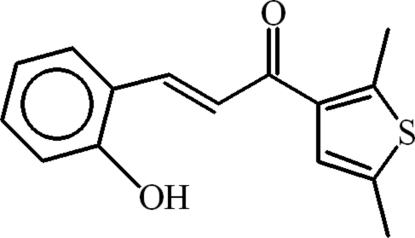


## Experimental

### 

#### Crystal data

C_15_H_14_O_2_S
*M* *_r_* = 258.32
Triclinic, 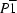

*a* = 7.6095 (3) Å
*b* = 7.7900 (3) Å
*c* = 12.3109 (7) Å
α = 98.527 (2)°
β = 91.943 (2)°
γ = 115.551 (1)°
*V* = 647.19 (5) Å^3^
*Z* = 2
Mo *K*α radiation
μ = 0.24 mm^−1^
*T* = 296 K
0.30 × 0.24 × 0.22 mm

#### Data collection

Bruker Kappa APEXII CCD diffractometer
Absorption correction: multi-scan (*SADABS*; Bruker, 2005 ▶) *T*_min_ = 0.968, *T*_max_ = 0.985
11156 measured reflections
3174 independent reflections
2720 reflections with *I* > 2σ(*I*)
*R*_int_ = 0.022

#### Refinement

*R*[*F* ^2^ > 2σ(*F* ^2^)] = 0.038
*wR*(*F* ^2^) = 0.112
*S* = 1.05
3174 reflections
166 parameters
H-atom parameters constrained
Δρ_max_ = 0.30 e Å^−3^
Δρ_min_ = −0.23 e Å^−3^

### 

Data collection: *APEX2* (Bruker, 2009 ▶); cell refinement: *SAINT* (Bruker, 2009 ▶); data reduction: *SAINT*; program(s) used to solve structure: *SHELXS97* (Sheldrick, 2008 ▶); program(s) used to refine structure: *SHELXL97* (Sheldrick, 2008 ▶); molecular graphics: *ORTEP-3 for Windows* (Farrugia, 1997 ▶) and *PLATON* (Spek, 2009 ▶); software used to prepare material for publication: *WinGX* (Farrugia, 1999 ▶) and *PLATON*.

## Supplementary Material

Crystal structure: contains datablocks global, I. DOI: 10.1107/S1600536810031284/bq2228sup1.cif

Structure factors: contains datablocks I. DOI: 10.1107/S1600536810031284/bq2228Isup2.hkl

Additional supplementary materials:  crystallographic information; 3D view; checkCIF report

## Figures and Tables

**Table 1:** Hydrogen-bond geometry (Å, °) *Cg*2 is the centroid of the C1–C6 benzene ring.

*D*—H⋯*A*	*D*—H	H⋯*A*	*D*⋯*A*	*D*—H⋯*A*
O1—H1⋯O2^i^	0.82	1.8900	2.7067 (14)	174
C8—H8⋯O1	0.93	2.2400	2.8416 (17)	122
C15—H15*A*⋯*Cg*2^ii^	0.96	2.79	3.652 (2)	150

Symmetry codes: (i) 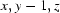; (ii) 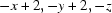.

**Fig. 1.**
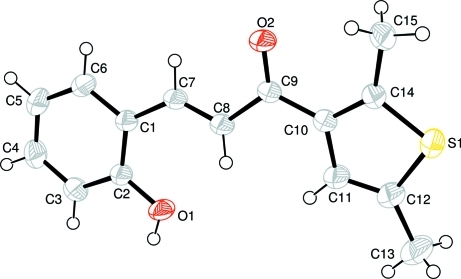
View of the title compound with the atom numbering scheme. The thermal displacements are drawn at the 50% probability level.

**Fig. 2.**
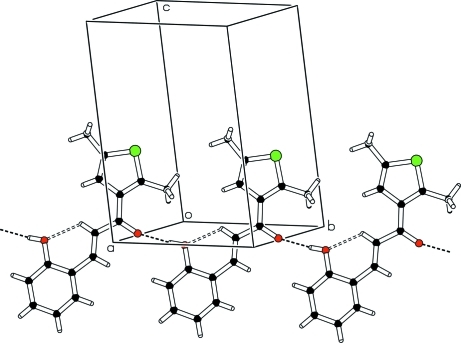
The partial packing (PLATON; Spek, 2009) which shows that molecules form polymeric chains extending along the b axis.

## supplementary crystallographic information

### Comment

An enone system between two aromatic rings is generally known as a chalcones. It is an important class of natural products which serve as precursors for the preparation of various flavonoids and exhibit interesting pharmacological activities (Mojzis *et al.*, 2008). Natural and synthetic chalcones have shown broad spectrum of biological activities such as anti-inflammatory (Vogel *et al.*, 2010), antituberculosis (Hans *et al.*, 2010), antifungal (Bandgar & Gawande, 2010), antimalarial (Domínguez *et al.*, 2001) and antileish-manicidal (Kayser & Kiderlen 2001). Due to wide application of chalcons in the present communication, we report the synthesis and crystal structure of title compound I (Fig. 1).

Recently we have reported the crystal structures of (II) *i.e.* (*E*)-1-(2,5-dimethyl-3-thienyl)-3-(2,4,5-trimethoxyphenyl)prop-2-en-1-one (Asiri *et al.*, 2010*a*) and (III) *i.e.* (2*E*)-3-(3,4-dimethoxyphenyl)-1-(2,5-dimethylthiophen-3-yl)prop-2-en-1-one (Asiri *et al.*, 2010*b*) which are related to the title compound and differ from (I) due to substitutions at the phenyl ring.

In (I), the group A (C1—C6/O1) of salicylaldehyde, the central group B (C7—C9/O2) and group C (C10—C15/S1) of 2,5-dimethylthiophen-3-yl moiety are planar with r. m. s. deviation of 0.0063, 0.0267 and 0.0100 Å, respectively. The dihedral angles between A/B, A/C and B/C are 1.20 (9), 8.46 (8) and 8.27 (9)°, respectively. In the title compound, an S(6) ring motif (Bernstein *et al.*, 1995) is formed due to intramolecular H-bonding of C—H···O type (Table 1, Fig. 2). The title compound is stabilized in the form of polymeric chains extending along the *b* axis due to O—H···O type of intermolecular H-bonding (Table 1, Fig. 2). The C—H···π (Table 1) and π–π interactions between the centroids of phenyl and heterocyclic rings at a distance of 3.7691 (9) Å [symmetry code: 1 - *x*, 1 - *y*, - *z*] also play important role in stabilizing the molecules.

### Experimental

A solution of 3-acetyl-2,5-dimethylthiophene (0.38 g, 2.5 mmol) and salicylaldehyde (0.30 g, 2.5 mmol) in an ethanolic solution of NaOH (3.0 g in 10 ml of ethanol) was stirred for 16 h at room temperature. The solution was poured into ice-cold water of pH = 2 (pH adjusted by HCl). The solid was separated and dissolved in CH_2_Cl_2_, this solution was washed with a saturated solution of NaHCO_3_ and then evaporated to dryness. The residue was recrystallized from methanol/chloroform.Yellow solid: Yield: 78%; m.p. 418–419 K.

### Refinement

The H-atoms were positioned geometrically (O—H = 0.86, C–H = 0.93–0.96 Å) and refined as riding with *U*_iso_(H) = x*U*_eq_(C, O), where x = 1.5 for hydroxy & methyl and x = 1.2 for aryl H-atoms.

### Crystal data

**Table:**

C_15_H_14_O_2_S	*Z* = 2
*M**_r_* = 258.32	*F*(000) = 272
Triclinic, *P*1	*D*_x_ = 1.326 Mg m^−^^3^
Hall symbol: -P 1	Mo *K*α radiation, λ = 0.71073 Å
*a* = 7.6095 (3) Å	Cell parameters from 2182 reflections
*b* = 7.7900 (3) Å	θ = 2.5–25.3°
*c* = 12.3109 (7) Å	µ = 0.24 mm^−^^1^
α = 98.527 (2)°	*T* = 296 K
β = 91.943 (2)°	Prism, yellow
γ = 115.551 (1)°	0.30 × 0.24 × 0.22 mm
*V* = 647.19 (5) Å^3^	

### Data collection

**Table:**

Bruker Kappa APEXII CCD diffractometer	3174 independent reflections
Radiation source: fine-focus sealed tube	2720 reflections with *I* > 2σ(*I*)
graphite	*R*_int_ = 0.022
Detector resolution: 8.10 pixels mm^-1^	θ_max_ = 28.6°, θ_min_ = 3.0°
ω scans	*h* = −10→11
Absorption correction: multi-scan (*SADABS*; Bruker, 2005)	*k* = −9→10
*T*_min_ = 0.968, *T*_max_ = 0.985	*l* = −16→16
11156 measured reflections	

### Refinement

**Table:**

Refinement on *F*^2^	Primary atom site location: structure-invariant direct methods
Least-squares matrix: full	Secondary atom site location: difference Fourier map
*R*[*F*^2^ > 2σ(*F*^2^)] = 0.038	Hydrogen site location: inferred from neighbouring sites
*wR*(*F*^2^) = 0.112	H-atom parameters constrained
*S* = 1.05	*w* = 1/[σ^2^(*F*_o_^2^) + (0.0593*P*)^2^ + 0.1362*P*] where *P* = (*F*_o_^2^ + 2*F*_c_^2^)/3
3174 reflections	(Δ/σ)_max_ = 0.001
166 parameters	Δρ_max_ = 0.30 e Å^−^^3^
0 restraints	Δρ_min_ = −0.23 e Å^−^^3^

### Special details

**Table:**

Geometry. Bond distances, angles *etc*. have been calculated using the rounded fractional coordinates. All su's are estimated from the variances of the (full) variance-covariance matrix. The cell e.s.d.'s are taken into account in the estimation of distances, angles and torsion angles
Refinement. Refinement of *F*^2^ against ALL reflections. The weighted *R*-factor *wR* and goodness of fit *S* are based on *F*^2^, conventional *R*-factors *R* are based on *F*, with *F* set to zero for negative *F*^2^. The threshold expression of *F*^2^ > σ(*F*^2^) is used only for calculating *R*-factors(gt) *etc*. and is not relevant to the choice of reflections for refinement. *R*-factors based on *F*^2^ are statistically about twice as large as those based on *F*, and *R*- factors based on ALL data will be even larger.

### Fractional atomic coordinates and isotropic or equivalent isotropic displacement parameters (Å^2^)

**Table:**

	*x*	*y*	*z*	*U*_iso_*/*U*_eq_	
S1	0.67628 (6)	1.06807 (5)	0.38493 (3)	0.0475 (1)	
O1	0.75707 (17)	0.38399 (14)	−0.02745 (8)	0.0464 (3)	
O2	0.73309 (16)	1.04466 (13)	0.01642 (8)	0.0461 (3)	
C1	0.79715 (18)	0.57560 (16)	−0.16540 (10)	0.0329 (3)	
C2	0.78885 (19)	0.40687 (17)	−0.13253 (10)	0.0343 (3)	
C3	0.8126 (2)	0.26851 (19)	−0.20796 (12)	0.0434 (4)	
C4	0.8396 (3)	0.2929 (2)	−0.31564 (12)	0.0504 (5)	
C5	0.8450 (3)	0.4557 (2)	−0.35080 (12)	0.0507 (5)	
C6	0.8256 (2)	0.5950 (2)	−0.27561 (11)	0.0427 (4)	
C7	0.77922 (19)	0.73111 (17)	−0.09244 (10)	0.0343 (3)	
C8	0.7412 (2)	0.74553 (17)	0.01224 (11)	0.0370 (3)	
C9	0.72983 (18)	0.92090 (16)	0.06888 (10)	0.0334 (3)	
C10	0.71496 (18)	0.94215 (17)	0.18852 (10)	0.0337 (3)	
C11	0.7304 (2)	0.81420 (19)	0.25716 (11)	0.0429 (4)	
C12	0.7131 (2)	0.8629 (2)	0.36472 (12)	0.0478 (4)	
C13	0.7182 (3)	0.7610 (3)	0.45856 (15)	0.0714 (7)	
C14	0.68407 (19)	1.08881 (17)	0.24864 (10)	0.0360 (4)	
C15	0.6562 (3)	1.2516 (2)	0.21248 (12)	0.0484 (5)	
H1	0.75495	0.28106	−0.01816	0.0696*	
H3	0.81024	0.15882	−0.18542	0.0520*	
H4	0.85434	0.19895	−0.36531	0.0605*	
H5	0.86144	0.47102	−0.42384	0.0609*	
H6	0.83168	0.70550	−0.29889	0.0513*	
H7	0.79737	0.83780	−0.12429	0.0411*	
H8	0.72160	0.64484	0.04996	0.0445*	
H11	0.75059	0.70703	0.22980	0.0515*	
H13A	0.73513	0.64780	0.43117	0.1070*	
H13B	0.59731	0.72392	0.49126	0.1070*	
H13C	0.82534	0.84640	0.51317	0.1070*	
H15A	0.77811	1.34346	0.19316	0.0725*	
H15B	0.61341	1.31403	0.27168	0.0725*	
H15C	0.55936	1.20163	0.14942	0.0725*	

### Atomic displacement parameters (Å^2^)

**Table:**

	*U*^11^	*U*^22^	*U*^33^	*U*^12^	*U*^13^	*U*^23^
S1	0.0656 (3)	0.0486 (2)	0.0318 (2)	0.0290 (2)	0.0077 (2)	0.0051 (1)
O1	0.0804 (7)	0.0384 (5)	0.0371 (5)	0.0376 (5)	0.0187 (5)	0.0171 (4)
O2	0.0761 (7)	0.0373 (5)	0.0389 (5)	0.0353 (5)	0.0124 (5)	0.0139 (4)
C1	0.0395 (6)	0.0311 (5)	0.0311 (6)	0.0176 (5)	0.0049 (5)	0.0075 (4)
C2	0.0425 (7)	0.0312 (5)	0.0321 (6)	0.0183 (5)	0.0060 (5)	0.0075 (4)
C3	0.0581 (8)	0.0336 (6)	0.0423 (7)	0.0244 (6)	0.0073 (6)	0.0047 (5)
C4	0.0663 (10)	0.0471 (8)	0.0384 (7)	0.0292 (7)	0.0063 (7)	−0.0037 (6)
C5	0.0690 (10)	0.0560 (8)	0.0285 (6)	0.0291 (7)	0.0091 (6)	0.0061 (6)
C6	0.0581 (8)	0.0422 (7)	0.0332 (7)	0.0250 (6)	0.0081 (6)	0.0126 (5)
C7	0.0435 (7)	0.0298 (5)	0.0353 (6)	0.0201 (5)	0.0061 (5)	0.0101 (4)
C8	0.0528 (7)	0.0299 (5)	0.0358 (6)	0.0238 (5)	0.0077 (5)	0.0094 (5)
C9	0.0413 (6)	0.0285 (5)	0.0343 (6)	0.0185 (5)	0.0044 (5)	0.0075 (4)
C10	0.0399 (6)	0.0301 (5)	0.0335 (6)	0.0174 (5)	0.0046 (5)	0.0065 (4)
C11	0.0595 (8)	0.0399 (6)	0.0379 (7)	0.0280 (6)	0.0075 (6)	0.0124 (5)
C12	0.0636 (9)	0.0472 (7)	0.0384 (7)	0.0276 (7)	0.0067 (6)	0.0145 (6)
C13	0.1111 (16)	0.0748 (12)	0.0457 (9)	0.0510 (11)	0.0156 (10)	0.0288 (8)
C14	0.0428 (7)	0.0336 (6)	0.0329 (6)	0.0186 (5)	0.0030 (5)	0.0044 (5)
C15	0.0726 (10)	0.0449 (7)	0.0412 (7)	0.0395 (7)	0.0063 (7)	0.0046 (6)

### Geometric parameters (Å, °)

**Table:**

S1—C12	1.7228 (16)	C11—C12	1.349 (2)
S1—C14	1.7099 (13)	C12—C13	1.504 (2)
O1—C2	1.3476 (16)	C14—C15	1.498 (2)
O2—C9	1.2298 (15)	C3—H3	0.9300
O1—H1	0.8200	C4—H4	0.9300
C1—C6	1.4011 (18)	C5—H5	0.9300
C1—C7	1.4564 (18)	C6—H6	0.9300
C1—C2	1.4083 (17)	C7—H7	0.9300
C2—C3	1.393 (2)	C8—H8	0.9300
C3—C4	1.377 (2)	C11—H11	0.9300
C4—C5	1.384 (2)	C13—H13A	0.9600
C5—C6	1.381 (2)	C13—H13B	0.9600
C7—C8	1.3295 (18)	C13—H13C	0.9600
C8—C9	1.4777 (18)	C15—H15A	0.9600
C9—C10	1.4713 (17)	C15—H15B	0.9600
C10—C11	1.4372 (19)	C15—H15C	0.9600
C10—C14	1.3788 (19)		
			
S1···C4^i^	3.686 (2)	C8···H11	2.7400
S1···H4^ii^	3.1500	C9···H15C	3.0600
O1···O2^iii^	2.7067 (14)	C9···H1^iv^	3.0800
O1···C8	2.8416 (17)	C11···H8	2.6800
O1···C15^iii^	3.2790 (18)	C15···H1^iv^	2.9800
O1···C9^i^	3.3986 (19)	H1···O2^iii^	1.8900
O2···O1^iv^	2.7067 (14)	H1···C9^iii^	3.0800
O2···C3^iv^	3.4160 (17)	H1···C15^iii^	2.9800
O2···C15	2.919 (2)	H1···H3	2.2700
O2···C7^v^	3.3745 (19)	H1···H15A^iii^	2.5600
O1···H15A^iii^	2.7900	H1···H15C^iii^	2.5800
O1···H15C^iii^	2.8800	H3···O2^iii^	2.7600
O1···H8	2.2400	H3···H1	2.2700
O2···H1^iv^	1.8900	H4···S1^vii^	3.1500
O2···H3^iv^	2.7600	H6···H7	2.3200
O2···H15A	2.8300	H7···O2	2.4000
O2···H15C	2.6200	H7···H6	2.3200
O2···H7	2.4000	H7···H15C^vi^	2.6000
O2···H15C^vi^	2.7700	H8···O1	2.2400
C2···C10^i^	3.583 (2)	H8···C2	2.9000
C3···O2^iii^	3.4160 (17)	H8···C11	2.6800
C3···C14^i^	3.559 (2)	H8···H11	2.1800
C4···S1^i^	3.686 (2)	H11···C8	2.7400
C6···C14^v^	3.442 (2)	H11···H8	2.1800
C7···C9^v^	3.5138 (19)	H11···H13A	2.5900
C7···O2^v^	3.3745 (19)	H13A···H11	2.5900
C8···O1	2.8416 (17)	H15A···O1^iv^	2.7900
C9···O1^i^	3.3986 (19)	H15A···O2	2.8300
C9···C7^v^	3.5138 (19)	H15A···H1^iv^	2.5600
C10···C2^i^	3.583 (2)	H15A···C1^v^	3.0600
C14···C3^i^	3.559 (2)	H15A···C5^v^	3.0500
C14···C6^v^	3.442 (2)	H15A···C6^v^	2.9600
C15···O2	2.919 (2)	H15C···O1^iv^	2.8800
C15···O1^iv^	3.2790 (18)	H15C···O2	2.6200
C1···H15A^v^	3.0600	H15C···C9	3.0600
C2···H8	2.9000	H15C···H1^iv^	2.5800
C5···H15A^v^	3.0500	H15C···O2^vi^	2.7700
C6···H15A^v^	2.9600	H15C···C7^vi^	2.9200
C7···H15C^vi^	2.9200	H15C···H7^vi^	2.6000
			
C12—S1—C14	93.47 (7)	C2—C3—H3	120.00
C2—O1—H1	109.00	C4—C3—H3	120.00
C2—C1—C7	124.39 (11)	C3—C4—H4	120.00
C6—C1—C7	118.02 (11)	C5—C4—H4	120.00
C2—C1—C6	117.59 (12)	C4—C5—H5	121.00
O1—C2—C3	121.65 (12)	C6—C5—H5	121.00
C1—C2—C3	120.04 (12)	C1—C6—H6	119.00
O1—C2—C1	118.31 (12)	C5—C6—H6	119.00
C2—C3—C4	120.50 (13)	C1—C7—H7	115.00
C3—C4—C5	120.73 (15)	C8—C7—H7	115.00
C4—C5—C6	118.88 (14)	C7—C8—H8	120.00
C1—C6—C5	122.23 (13)	C9—C8—H8	120.00
C1—C7—C8	130.57 (12)	C10—C11—H11	123.00
C7—C8—C9	120.25 (11)	C12—C11—H11	123.00
O2—C9—C10	121.58 (12)	C12—C13—H13A	109.00
C8—C9—C10	118.20 (11)	C12—C13—H13B	109.00
O2—C9—C8	120.22 (11)	C12—C13—H13C	109.00
C9—C10—C14	123.69 (11)	H13A—C13—H13B	109.00
C11—C10—C14	111.61 (11)	H13A—C13—H13C	110.00
C9—C10—C11	124.70 (12)	H13B—C13—H13C	109.00
C10—C11—C12	114.24 (13)	C14—C15—H15A	109.00
S1—C12—C13	121.37 (12)	C14—C15—H15B	109.00
C11—C12—C13	128.61 (15)	C14—C15—H15C	109.00
S1—C12—C11	110.00 (11)	H15A—C15—H15B	109.00
S1—C14—C15	118.86 (10)	H15A—C15—H15C	109.00
C10—C14—C15	130.46 (12)	H15B—C15—H15C	110.00
S1—C14—C10	110.68 (10)		
			
C14—S1—C12—C11	0.02 (16)	C4—C5—C6—C1	−1.2 (3)
C14—S1—C12—C13	−178.73 (15)	C1—C7—C8—C9	−179.80 (15)
C12—S1—C14—C10	−0.21 (12)	C7—C8—C9—O2	8.3 (2)
C12—S1—C14—C15	179.18 (14)	C7—C8—C9—C10	−171.60 (14)
C6—C1—C2—O1	−178.51 (14)	O2—C9—C10—C11	−173.33 (14)
C6—C1—C2—C3	1.3 (2)	O2—C9—C10—C14	6.7 (2)
C7—C1—C2—O1	1.8 (2)	C8—C9—C10—C11	6.6 (2)
C7—C1—C2—C3	−178.36 (14)	C8—C9—C10—C14	−173.36 (14)
C2—C1—C6—C5	0.1 (2)	C9—C10—C11—C12	179.67 (14)
C7—C1—C6—C5	179.74 (16)	C14—C10—C11—C12	−0.37 (19)
C2—C1—C7—C8	−4.6 (3)	C9—C10—C14—S1	−179.69 (11)
C6—C1—C7—C8	175.77 (16)	C9—C10—C14—C15	1.0 (3)
O1—C2—C3—C4	178.23 (16)	C11—C10—C14—S1	0.36 (16)
C1—C2—C3—C4	−1.6 (2)	C11—C10—C14—C15	−178.95 (16)
C2—C3—C4—C5	0.4 (3)	C10—C11—C12—S1	0.21 (18)
C3—C4—C5—C6	0.9 (3)	C10—C11—C12—C13	178.83 (17)

Symmetry codes: (i) −*x*+1, −*y*+1, −*z*; (ii) *x*, *y*+1, *z*+1; (iii) *x*, *y*−1, *z*; (iv) *x*, *y*+1, *z*; (v) −*x*+2, −*y*+2, −*z*; (vi) −*x*+1, −*y*+2, −*z*; (vii) *x*, *y*−1, *z*−1.

### Hydrogen-bond geometry (Å, °)

**Table:**

Cg2 is the centroid ofthe C1–C6 phenyl ring.

**Table:**

*D*—H···*A*	*D*—H	H···*A*	*D*···*A*	*D*—H···*A*
O1—H1···O2^iii^	0.82	1.8900	2.7067 (14)	174
C8—H8···O1	0.93	2.2400	2.8416 (17)	122
C15—H15A···Cg2^v^	0.96	2.79	3.652 (2)	150

Symmetry codes: (iii) *x*, *y*−1, *z*; (v) −*x*+2, −*y*+2, −*z*.
